# From models to medicine: systematic development and evaluation of animal models for *Chlamydia psittaci* respiratory and genital tract infections

**DOI:** 10.3389/fimmu.2026.1754621

**Published:** 2026-03-23

**Authors:** Jingyi Zhang, Hua Wu, Yuanzhi Li, Chuan Wang

**Affiliations:** 1 Institute of Pathogenic Biology, School of Basic Medicine, Hengyang Medical College, University of South China, Hunan Provincial Key Laboratory for Special Pathogens Prevention and Control, Hengyang, Hunan, China; 2 Clinical Microbiology Laboratory, Affiliated Hengyang Hospital of Hunan Normal University & Hengyang Central Hospital, Hengyang, Hunan, China

**Keywords:** *Chlamydia psittaci*, domestic animals, infection mechanism, mice and guinea pigs, avain and poultry, vaccine research

## Abstract

*Chlamydia psittaci* (*C. psittaci*) is a zoonotic pathogen causing severe respiratory infections and systemic complications. Animal models are a crucial approach for studying *C. psittaci*. They can simulate the natural infection process and reveal the interaction between the host and the pathogen. This review examines the roles and limitations of various animal models in understanding the infection mechanism, developing vaccines and therapies, and researching the evolution and transmission of the pathogen. Mouse and guinea pig models are widely used in immunological research because they have numerous advantages in terms of genetic manipulation, cost-effectiveness, and operability. Poultry, which are natural hosts of *C. psittaci*, are of great significance for the study of the pathogenic characteristics and transmission routes of this pathogen in birds. Compared with small rodents, the immune systems of large mammals, such as non-human primates, are more complex. This is because they have a closer physiological relationship with humans and are the gold standard for evaluating treatment efficacy. Due to the significant disparities in standardized cross-model comparison data, future efforts should focus on the following aspects. (a) Establishing a unified evaluation framework to assess respiratory and reproductive tract infections. (b) Leveraging new technologies such as tissue organ models and humanized mice to eliminate species-specific differences. (c) Prioritizing the conduct of preclinical trials based on non-human primates to ultimately bridge the critical gap between basic research and the development of effective response measures.

## 1 Introduction

In recent years, the increasing trend of human cases of psittacosis has become an important public health issue that cannot be ignored (1–5). *Chlamydia psittaci* (*C. psittaci*) is a pathogen that must reside within cells. It is the causative agent of psittacosis or avian disease, primarily causing fatal respiratory disorders in birds, but can also affect various mammals, including humans (6). Its host range is extremely wide, including seemingly harmless domestic poultry and ornamental birds, as well as economically valuable domestic animals. All these organisms can serve as natural hosts and asymptomatic carriers (7). Similar to *Coxiella burnetii*, *C. psittaci* is usually transmitted from animals to humans through various means, including direct contact with infected animal tissues or secretions, such as saliva, urine or feces, as well as inhalation of aerosols containing the bacteria (8). The symptoms of this infection are quite extensive, ranging from no symptoms to being fatal. It can manifest as mild conditions such as colds, coughs and runny noses, or severe ones like pneumonia, breathing difficulties, and multiple organ failure (9). The diversity and concealment of these symptoms pose significant challenges for clinical diagnosis and timely intervention. However, our understanding of the immune mechanism underlying its pathogenesis is still far from complete, and currently there are no effective vaccines or precise, efficient targeted drugs available. Solving this core contradiction largely depends on whether we can recreate and deeply analyze the interaction between the pathogen and the host in a controlled laboratory environment. This underscores the central importance of appropriate animal models for studying *C. psittaci* pathogenesis and interventions.

All *Chlamydia* are obligate intracellular parasites, possessing unique biphasic life characteristics. They are capable of overcoming numerous obstacles, such as entering host cells, persisting and reproducing within host cells, and surviving in harsh extracellular environments (10). Two forms that are different in both function and appearance will alternate in a certain sequence. The elementary body (EB) is infectious and in a metabolic quiescent state, responsible for attaching to the target host cell and facilitating its entry into the cell; while the reticular body (RB) is an unfinished, non-infectious and metabolically active intracellular form, which replicates by binary fission and is reorganized into the basic body before being released through cell cycle (11, 12). However, the pathogenic core of *C. psittaci* lies in the host immunopathological response it triggers, rather than direct cytotoxicity (13). The severity of the disease is closely related to excessive natural immune responses, imbalances in T cell reactions, such as Th1/Th17 types, and associated cytokine storms (14–17). Therefore, an ideal animal model must be capable of accurately replicating this complex immune network, so as to study the balance mechanism between the protective clearance function of the immune system and its destructive responses.

Due to ethical constraints and the potential risks of this pathogen, a comprehensive study of the human *C. psittaci* infection process cannot be conducted. Therefore, animal models must be utilized to study the pathogenesis and evaluate intervention measures. This review focuses on the most common infection models of psittacosis, such as those using mice and guinea pigs as research subjects. In addition, non-human primates share a high degree of similarity with humans in terms of genetic evolution and physiological characteristics.

Therefore, it is necessary to use animal models to study its pathogenic mechanism and evaluate intervention measures. However, different animal models have their own advantages and limitations in simulating different aspects of human infection (18). The core themes of this review are to systematically compare non-natural hosts with natural hosts, emphasizing that such a comparison can provide new insights for the treatment of psittacosis. Through multi-dimensional comparisons and evaluate the ability of each model in recapitulating the key characteristics of human diseases, which cover various aspects such as initial immune activation, pathological progression, and transmission dynamics. Through a comprehensive analysis of the data, this review not only depicts the current situation but also identifies key knowledge gaps. Therefore, it provides a new perspective and immediate practicality for accelerating the formulation of effective measures to address this long-standing public health threat.

## 2 Non-natural host animal model

### 2.1 Mouse model

Due to the diversity of the genetic background of mouse models, the presence of specific defects, the relatively low cost, the ease of accessibility, and the availability of existing immunological reagents, it is widely believed that mice are the ideal small animal model for studying various pathogen infections (19). BALB/c and C57BL/6, as well as genetic reference populations like BXD, have played a crucial role in elucidating the pathogenesis and immune response of *C. psittaci*. These models are of great significance for clarifying the basic principles of immune mechanisms and for subsequent evaluation of new vaccine candidates and therapeutic interventions, thereby closely linking basic research with translational development (20, 21).

#### 2.1.1 BALB/C mouse model

The BALB/c mouse is widely used in immunological research as an infection model and can also serve as a preclinical assessment tool for drugs and vaccines (22–24). Similar to psittacosis infection, the BALB/c mouse strain is susceptible to *Salmonella* and other pathogens and develops persistent infections, while the Th2-type immune response triggered simultaneously provides a unique perspective for studying the balance between immune protection and immune pathology (25–27).

BALB/C mice are typically used to evaluate the efficacy of vaccines. Many pathogen vaccines are safe when administered to BALB/c mice via a single intramuscular injection or nasal administration and can trigger strong humoral and cellular immune responses (28, 29). The research has found that the level of specific antibodies induced by the vaccine and the intensity of T-cell responses are positively correlated with the protective efficacy of the vaccine, providing reliable indicators for the immunological assessment of the vaccine (30, 31). In addition, BALB/C mice can effectively simulate vaccine-mediated immune memory responses, providing an important platform for studying long-term protective effects.

The value of BALB/c mice cannot be ignored in the field of pathogenic mechanisms research. After infection, BALB/c mice induce a dominant Th2-type immune response, with exhibiting a higher liver-to-body weight and spleen-to-body weight ratio compared to other inbred mice (32). In tissues, microscopic lesions are represented by infiltrates of lymphocytes, neutrophils and plasma cells (33, 34). Notably, BALB/c mice tend to have distinct immunopathological features following infection, including excessive inflammatory responses and tissue damage, providing important clues to the mechanism of immune-mediated injury induced by *C. psittaci* infection.

The unique advantages of BALB/c mice in the study are reflected mainly in several aspects. 1) Its susceptibility to *C. psittaci* makes disease modeling more reliable and reproduces the full course of the disease from acute to chronic infections. 2) The strong immune response characteristics exhibited by this strain, both protective and pathological, significantly enhance the observability and reproducibility of the experiments. 3) Its definite Th2-type immune bias provides an ideal model for studying the association of specific immune phenotypes with disease outcomes.

#### 2.1.2 C57BL/6 mouse model

The most commonly used strain is the C57BL/6 mice, as many transgenic and knockout animals are maintained on this genetic background. Its strong genetic manipulation background and characteristic immune response patterns make it an ideal model system for exploring the molecular mechanisms of host-pathogen interactions.

We know that the C57BL/6 mouse has a rich collection of gene knockout, transgenic and conditional knockout strains. This function enables researchers to precisely analyze the functions of specific genes during the process of *C. psittaci* infection. The studies conducted using IFN-γ gene-deficient mice clearly demonstrated the crucial role of this cytokine in controlling the replication process of pathogens (35) and further elucidated its mechanism of action by blocking the relevant pathways (36).

Furthermore, C57BL/6 mice exhibited a strong Th1-type immunologic bias (37, 38). After infection, high levels of IFN-γ and IL-12 can be rapidly produced, and this will trigger a strong IgG2c antibody response. This immune characteristic gives it extremely strong resistance to *C. psittaci*. The pathogen can be quickly eliminated and the infection process is relatively mild. However, this powerful immune response can also lead to immune pathological damage, especially in cases of high-dose infection or under specific genetic modification conditions. This provides an important research window for studying the mechanism of immune-mediated tissue damage.

C57BL/6 mice are usually infected by intranasal inoculation, and the required infection dose varies depending on the toxicity of the strain (39). He transformed the acute infection into a chronic infection and observed pathological features such as inflammatory cell infiltration in the lungs, thickening of the alveolar walls, and local lymphoid tissue hyperplasia (40, 41). However, the strong anti-infection ability of C57BL/6 mice may affect the assessment of the pathogenicity of the pathogen, and their specific immune background may not fully simulate the complexity of human infections.

The different immune phenotypes of BALB/c and C57BL/6 mice (**Table 1**) are not merely experimental observations but are determined by profound genetic factors. The Th1-biased C57BL/6 strain usually exhibits a more effective clearance response triggered by IFN-γ, while the Th2-biased BALB/c mice often show more obvious immunopathological changes and chronic manifestations (32, 42). Therefore, the selection among these strains is significant. BALB/c mice are ideal models for studying the immune pathology induced by vaccines and the mechanisms of persistent infections, while C57BL/6 mice (especially their numerous genetically modified derivatives) are unrivaled in analyzing the cell-mediated immunity and resistance molecular pathways.

**Table 1:** A comparative summary of mouse models for *C. psittaci* infection.

Feature	BALB/c Model	C57BL/6 Model
Primary application	vaccine efficacy immunopathology chronic infection	molecular mechanisms of immunity gene function resistance
Immune bias	Th2-biased	Th1-biased
Immune mechanisms	IL-4 leads to Th2 cells that express GATA-3 and secrete IL-4, IL-5 and IL-13.	IL-12 promotes the development of Th1 cells that express the transcription factor T-bet (Tbx21) and produce (IFN)-gamma,
Infection course	prone to establishing persistent infection; longer disease duration	acute and self-limiting; rapid pathogen clearance
Pathological features	more significant immunopathological damage; increased liver/spleen-to-body weight ratios; prominent infiltration of lymphocytes and plasma cells	relatively milder pathological damage; primarily characterized by pulmonary inflammatory cell infiltration and alveolar wall thickening

The BALB/c mice, due to its tendency toward Th2-type immune responses, provides a powerful system for simulating the progression of persistent infections and for vaccine-induced immunity in susceptible hosts. Its pathological features, characterized by significant lymphoid tissue infiltration and systemic effects, offer a basis for the reliable reproduction of chronic disease components. In contrast, the C57BL/6 mice, due to its inherent Th1 bias and stronger resistance, become a powerful model for dissecting the molecular mechanisms of protective cell-mediated immunity. When utilized as the genetic background for complex gene targeting approaches, its greatest application lies in the ability to precisely validate the functions of specific host factors, such as cytokines and signaling molecules.

#### 2.1.3 BXD mouse model

By crossing two C57BL/6 and DBA/2 strains with distinct genetic backgrounds, the BXD recombinant strain was developed. It can simulate the genetic heterogeneity observed in complex human diseases (43).

Genetic variations in immune-related GTPases are the key factor determining the susceptibility of BXD to pathogens (44). The *Ctrq3* gene locus located on chromosome 11 serves as the core genetic basis, effectively inhibiting intracellular pathogen replication by regulating the expression of such GTPases. The polymorphism of this gene locus does not directly control the levels of pro-inflammatory cytokines but rather affects the disease outcome by regulating the activation state of macrophages (45). Additionally, studies have found that macrophage function plays a dual protective role, enhancing its antibacterial ability and inhibiting pathological infiltration of neutrophils (45). Experimental evidence shows that the absence of macrophages leads to excessive accumulation of neutrophils and aggravated tissue damage, highlighting its central role in limiting immune pathological damage (46).

The BXD recombinant inbred strain mouse model overcomes the genetic limitations of traditional purebred strains and can better simulate the genetic diversity of human populations. This model not only confirms that disease outcomes are regulated by the dual effects of pathogen virulence and host genes but also reveals the existence of independent genetic pathways in the host genome that regulate antibacterial defense and immune pathological damage, providing a new perspective for understanding the mechanism by which host genetic background affects infection outcomes through regulating the balance of innate immunity.

### 2.2 Guinea pig model

As a classic model, guinea pigs hold a unique position in the study of *C. psittaci*. This position is determined by the critical balance between their physiological similarity to humans and the practical limitations of the research. The high susceptibility of guinea pigs to the pathogen not only ensures the efficiency of infection establishment but also helps to reproduce the core clinical features, especially the fever response (47). The physiological similarity of guinea pigs to humans in terms of thermoregulation provides clinically relevant indicators for disease progression and treatment monitoring. Moreover, the complement system and antibody response patterns of guinea pigs are also more closely related to those of humans in evolution (48). This immunological similarity provides a more predictive framework for evaluating the protective immunity induced by vaccines, especially for those measures that rely on the mechanisms of opsonization and complement-dependent responses. However, the guinea pig model also has some obvious limitations. Due to the scarcity of immunological reagents and molecular tools, as well as the lack of a complete annotated sequence of the guinea pig genome, this has greatly hindered a comprehensive analysis of the host response in this model (49).

The guinea pigs also exhibited typical pathological features, such as conjunctival scarring and eyelid deformities, which were highly consistent with the pathological process of *C. trachomatis (*50, 51). During the chronic stage, a significant increase in the number of monocytes also provides an important perspective for the study of the immune mechanism. In addition, their reproductive physiology and estrous cycle are similar to those of humans, making them suitable as a translational model for studying chlamydia between mice and humans (52). The reproductive tract infections in guinea pigs can naturally simulate the characteristics of human *chlamydia trachomatis* infections (53). These findings all indicate that they can bridge the gap between acute infection and long-term immune pathological consequences. In conclusion, the guinea pig model is uniquely capable of being used to study the chronic infection process as well as the sequelae of the reproductive system.

### 2.3 Non-human primate model

In mouse models, interfering with specific immune pathways often leads to resistance and alleviation of the disease. However, in human infected individuals, severe cases often coexist with a strong Th1 response or inflammation. This contradiction suggests that human severe cases may not be caused by insufficient Th1 response, but rather by excessive or dysregulated Th1 response or the existence of key immune pathological pathways different from those in mice (such as the excessive activation of specific inflammasomes). To understand the immune etiology of human severe cases, in-depth research should be conducted in non-human primates(NHPs) that are more closely related to the human physiology and immune system (54).

The unique manifestations of immune dysfunction related to age in non-human primates have a crucial impact on the infection outcome, indicating consistency in the research on immune aging (55). NHPs are important in drug and vaccine evaluations against filamentous viruses such as *Ebola* because they can faithfully reproduce human disease processes (56). This principle also applies to other important infectious diseases, such as *C. psittaci* infection, especially when studying severe systemic infections and pneumonia.

However, the high costs, strict ethical considerations, and the lack of genetic tools specific to certain species make it difficult to conduct in-depth mechanism research. The strategic value of non-human primates lies not in exploratory research, but in answering key, targeted and transformative questions. Their best use is to validate discoveries and study complex human-specific disease manifestations.

## 3 Natural host animal model

Natural host models have unique value in *C. psittaci* research. Compared to laboratory animal models, they can truly reproduce the natural infection process and transmission dynamics of pathogens. Studies have shown that the number of pathogens required for *leishmaniasis* transmission by gerbils as natural hosts is much lower than that of experimental mice, showing higher fidelity of the natural host model in the simulated transmission link (57). However, such models have limitations such as high cost, inapparent clinical symptoms, and complex genetic background. At present, it is mainly applied in research on pathogen ecology and transmission mechanism and provides an important platform for vaccine potency evaluation. We should combine multi-omics integrative analysis. with artificial intelligence methods to promote the standardization of models and strengthen their translational application in the prevention and control of zoonoses.

### 3.1 Bovine model

Compared to small animals such as rodents, cattle models are closer to humans in multiple aspects, such as anatomy, metabolism, and physiology. The bovine model is used to validate candidate vaccines and anti-chlamydia drugs that show potential in small animals such as mice, and its improved immune system provides more reliable data on immunogenicity and protective efficacy (58). The model well simulates the natural infection process. Acute symptoms peak 2–4 days after inoculation, accompanied by a natural immune response predominantly in neutrophils and a systemic inflammatory response (59–61). In addition, the high similarity between cattle and humans in ovarian structure, follicular dynamics, and reproductive endocrine regulation makes the experimental data obtained from the cattle model more valuable in predicting human infection and immune responses (59, 62). This provides an important perspective for studying the comprehensive impact of *Chlamydia* on livestock production.

The bovine model also has its inherent limitations. The high breeding and experimental costs limit its large-scale application. And compared with mouse models with abundant genetic tools, the relative paucity of genetic manipulation tools in the bovine model has to some extent constrained its application in fine interpretation of gene functions. It undertakes and validates mechanistic findings obtained from small models such as mice and guinea pigs for preclinical efficacy assessment in a more human physiological system, providing critical and more predictive data support for subsequent non-human primate studies or clinical trials (58).

### 3.2 Sheep model

The bovine is more suitable for studies of acute respiratory infections and systemic diseases, while the sheep model has unique advantages in reproductive biology and in the study of chronic infections. They form a good complement. Sheep models demonstrate unique advantages in *C. psittaci* research, particularly in the fields of reproductive tract infections and chronic diseases. And the reproductive cycle of sheep is clear and easy to observe, making it an ideal model for studying chlamydial-related abortion and reproductive disorders. Studies have shown that placental inflammation and miscarriage occur in pregnant sheep after infection with *C. psittaci*, a pathological process similar to that seen in humans (63), and the sheep can more accurately study the infection dynamics at all stages of pregnancy.

The sheep shows unique value in the study of respiratory tract infections, its lung structure is highly similar to that of humans, which is convenient for carrying out bronchoalveolar lavage and other operations. This model not only simulates acute infection symptoms but also reproduces chronic infection processes, which is of great significance for studying persistent infection (64). Equally ruminant animals, such as cattle, the sheep model has a prominent advantage in mucosal immune research, as its respiratory mucosa structure is similar to that of humans, and it can induce the production of specific mucosal IgA antibodies after infection, providing an important basis for vaccine development (65, 66).

However, compared with mouse models, sheep have a more complex genetic background and relatively limited gene manipulation tools, which to some extent limits the depth of molecular mechanism research. Longer breeding cycle and higher experimental costs are also factors to consider.

### 3.3 Pig model

The pig model shows unique value in *C. psittaci* research, with potential in particular in reproductive tract infection and persistent infection research. Studies have shown that pathogens can infect pigs and cause typical reproductive system diseases, which are mainly manifested clinically as miscarriage, stillbirth and weak birth of pregnant sows (67, 68).

In the study of reproductive tract infections, pig models provide key insights. *Andreas Pospischil* et al. found that chlamydial abnormal structures were present in pigs, suggesting that the reproductive model of pigs may be suitable for studying persistent infection with *Chlamydia in vivo (*69). When pigs were infected with *C. trachomatis*, severe symptoms of salpingitis and endometritis were observed (68). This indicates that *C. psittaci* infection may interfere with the normal reproductive process by affecting the function of the fallopian tubes and the health of the uterus.

### 3.4 Horse model

The study detected *C. psittaci* in both the reproductive tract and environmental samples of horses, with a PCR positive rate of 21.6%. Some of the positive individuals had a history of reproductive system diseases such as miscarriage or embryo loss, suggesting that this form of psittacosis may be related to reproductive disorders and highlighting the value of horses as potential animal models for studying this bacterium. Additionally, the same genotype strains were detected in environmental samples, confirming the risk of cross-transmission within the horse population and demonstrating the crucial role of the environment in the spread of the pathogen (70). However, the number of relevant studies is limited, and there is a lack of standardized experimental infection procedures as well as systematic pathological descriptions. Therefore, future research should elucidate the colonization mechanism and pathogenic process of *C. psittaci* in the reproductive tract of horses.

### 3.5 Duck and chicken model

Ducks and chickens, as important natural hosts of *C. psittaci*, offer unique perspectives in the study of the natural transmission chain of this pathogen, the transmission risks between different species, and the immune responses of poultry.

Epidemiological data show that the infection rate of *C. psittaci* in duck flocks can reach 39% (71), highlighting its crucial role in the transmission and maintenance of this pathogen in the environment. Ducks are highly susceptible to this pathogen and can continuously excrete it through their feces, causing environmental pollution (72). Therefore, they have become an ideal model for studying environmental transmission and cross-infection. Moreover, the duck model has special value in exploring the impact of immunosuppression on infection. For example, infection with the *Tumbusu virus* (TMUV) can severely damage duck immune organs and inhibit vaccine-induced humoral immunity (73), suggesting that using this model to study the dynamics of chlamydia infection under immunosuppression conditions has great potential.

Compared with ducks, chickens are the most commonly used and more standardized poultry model in research, and they have an irreplaceable advantage in vaccine evaluation and immune mechanism studies. Their infection rate is approximately 13% (71). Although its genetic background is not as clear as that of ducks, its mature experimental system makes it the preferred choice for standardized research. *Zhou Jizhang* and his team successfully constructed a recombinant adenovirus vaccine containing the main outer membrane protein (MOMP) gene of *C. psittaci*. This vaccine can trigger strong humoral and cellular immune responses, with a protection rate of up to 80%-90% (74). In addition, chickens have a stronger HI antibody response to the H7N9 avian influenza vaccine than ducks (75). This species-specific immune response difference provides an important reference for optimizing vaccine strategies. The vascular zone (AV) and villous allantoic membrane (CAM) of chicken embryos also provide a unique platform for studying pathogen-host interactions (76).

### 3.6 Turkey model

The high susceptibility of turkeys to pathogens makes them an ideal natural host for studying acute infections and their transmission dynamics. Studies have shown that turkeys can exhibit severe systemic symptoms (77, 78). Its high sensitivity is conducive to the precise modeling of systemic infections and enables crucial research on transmission dynamics and airborne transmission (79). This makes the turkey an ecologically significant model for evaluating the effectiveness of vaccines.

The turkey model simulates the specific epidemiological conditions of poultry rather than directly predicting human diseases. Therefore, its practical value lies in its targeted application significance. Specifically, it is manifested in the following two points. 1) Analyzing the driving factors of poultry epidemics. 2) Testing the ability of vaccines to induce long-lasting immunity at the population level.

## 4 The differences between natural and non-natural host

**Figure 1:**
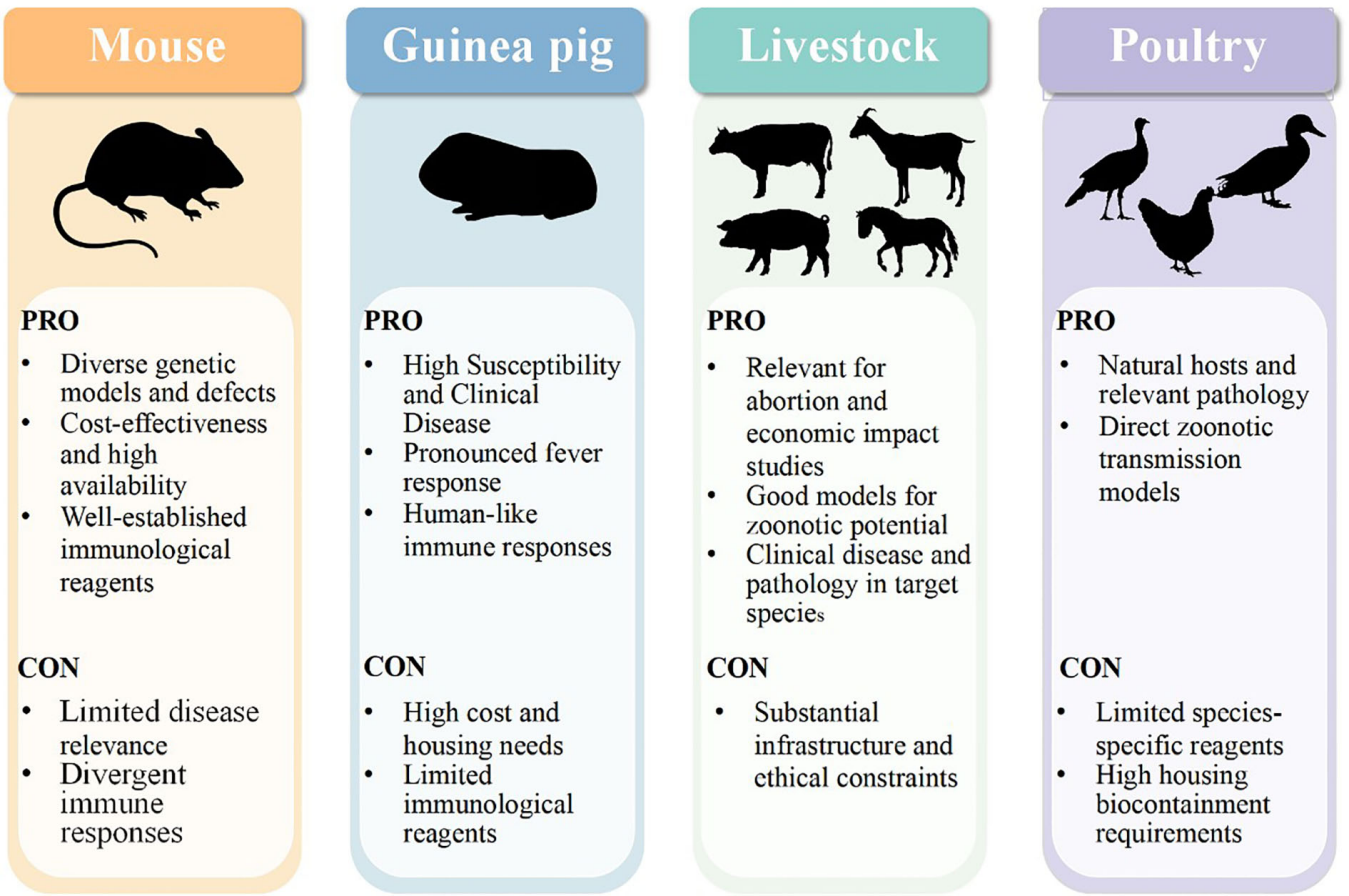
Comparative advantages and disadvantages of animal models in *C. psittaci* infection. The figure summarizes the advantages and disadvantages of the mouse, guinea pig, livestock, and poultry models to study respiratory and genital tract infection with *C. psittaci*. Mice are the preferred model for immune mechanism research due to their diverse genetic tools, mature immune reagents, and high cost-effectiveness. Guinea pigs, with their high susceptibility to pathogens and ability to reproduce typical clinical features such as fever, are ideal models for studying clinical symptoms and human-like immune responses. Livestock models are irreplaceable in researching abortion, economic impacts, and direct zoonotic transmission risks. Poultry models, as natural hosts, offer a unique perspective on revealing environmental transmission chains and cross-species transmission mechanisms. However, no single model can fully simulate the full complexity of human diseases. Mice have limitations such as limited disease relevance and species-specific immune responses; guinea pig and livestock models face practical challenges like high costs, strict housing requirements, or ethical constraints; poultry models are also limited by the lack of species-specific reagents.

This review classifies them into natural hosts and non-natural hosts based on their relationship with pathogens. And analyze their similarities and differences to provide guidance for basic research and clinical applications (**Figure 1**).

### 4.1 Infection and pathological features

*C. psittaci* is mainly infected by direct contact, inhalation of aerosols or dust containing pathogens, and by vertical transmission (63, 80). Natural host models, including poultry, sheep, pig, and cattle, show distinct chronic tendency and persistent bacterial excretion characteristics during infection. In the poultry model, turkeys were highly susceptible to *C. psittaci* and presented with acute systemic symptoms after infection, while the duck model was more suitable for studying environmental transmission dynamics (81). In a ruminant animal model, infection of the sheep reproductive tract persists for several years, presenting typical pathologic changes of placental inflammation and abortion, and the presence of pathogens can still be detected after abortion (63, 72). This model of chronic or persistent infection established in natural hosts is of great significance for studying the vertical transmission mechanism of pathogens and their impact on reproductive health (67, 68).

In contrast, non-natural hosts mimic human disease primarily by experimental infection. Commonly used methods include endotracheal inoculation and intraperitoneal injection. Among them, endotracheal inoculation can better simulate the natural route of respiratory infection. Symptomatic immune responses were observed in different experimental animals after infection. BALB/c and C57BL/6 mice rapidly developed immune responses and significant pulmonary inflammation after transtracheal infection, whereas C57BL/6 mice exhibited early defense against pathogen proliferation by neutrophils via the iNOS/NO pathway. While guinea pig models were prone to chronic infections, manifested by recurrent conjunctival inflammation and other lesions, providing a unique perspective on the mechanisms of persistent infection and immunological pathology.

### 4.2 Immune response pattern

The natural host model can better reflect the immune balance state during the infection process. For instance, in the case of continuous infection in the reproductive tract of sheep, there are chronic pathological features such as lymphocyte and plasma cell infiltration and tissue fibrosis, which are highly similar to the lesions observed in human infections (63). In the avian model, the mucosal immune system of ducks and chickens provides a unique window for local respiratory tract immune research (82), and the chicken model, due to its mature experimental system, demonstrates significant value in vaccine immune assessment (74). As an emerging natural host model, horses offer a new perspective for exploring the mechanisms of immune tolerance and persistent pathogen presence (70).

In contrast, the immune responses of non-natural host models exhibit significant species specificity. As a commonly used model, the immune responses of mice show a strain-dependent bias: the BALB/c strain tends to have a Th2-type immune response, while the C57BL/6 strain is more inclined towards a Th1-type (32, 37, 38). Although this specificity limits the direct application of these models to humans, it provides a controllable and convenient research platform for analyzing the mechanism of specific immune pathways.

### 4.3 Research application value

The natural host model holds an irreplaceable value in the study of infectious disease transmission dynamics. The avian model, due to its social behavior and environmental disinfection properties, has become an ideal platform for studying direct contact transmission and aerosol transmission (83). The long-term persistent infection characteristics of the sheep model provide a unique opportunity to study vertical transmission and chronic infections of the reproductive tract. In terms of mechanism interpretation and development of intervention strategies, non-natural host models demonstrate significant advantages. Mouse models play a central role in the study of host-pathogen interaction mechanisms with their abundant genetic tools and standardized operating procedures (84, 85). The guinea pig model is of great value in the immunogenicity evaluation of vaccines (48, 86).

**Figure 2:**
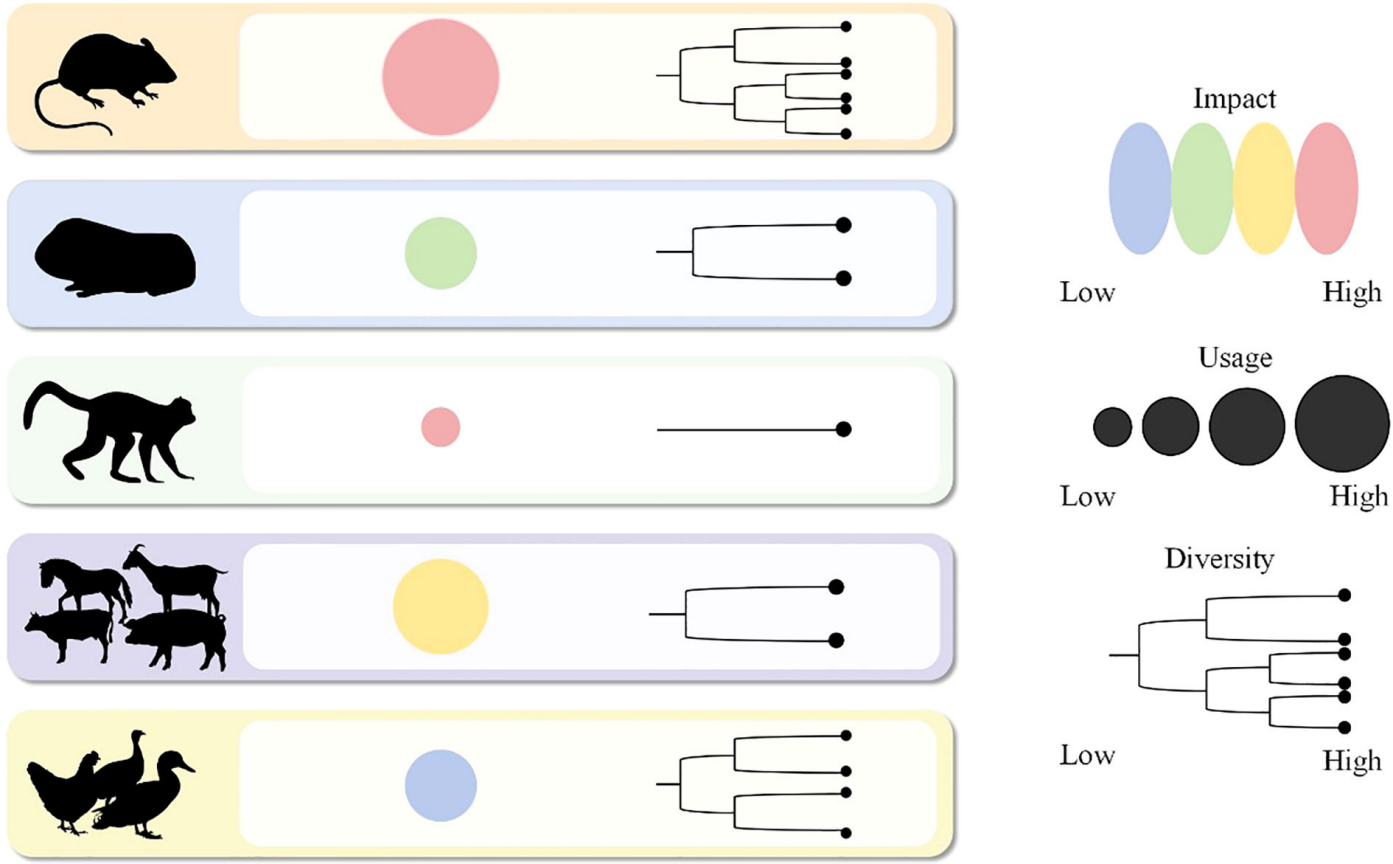
Overview of animal models for *C. psittaci.* This image demonstrates a simple framework for evaluating an animal model of *C. psittaci* infection. The framework considers three key dimensions. Impact, which refers to the severity of disease caused by infection. Usage refers to the commonness and data accumulation of the model in scientific research. Diversity, referring to the range and diversity of animal species used. Each dimension is graded as low to high. Based on this framework, the most appropriate animal model can be selected according to different experimental goals. For example, mouse models, particularly the C57BL/6 and BALB/c strains, exhibit the highest diversity in genetic tools and applications, enabling detailed mechanistic studies on immunity and pathogenesis. Guinea pig models, with their high physiological usage, provide critical insights into mucosal and chronic infections. Natural hosts such as poultry and livestock are invaluable for studying transmission dynamics and zoonotic potential. Among all, non-human primates demonstrate the greatest impact due to their unparalleled translational value for evaluating therapeutic and vaccine efficacy, closely mirroring human disease.

This review specifically mentions vaccine research here. Mouse models are used not only to evaluate the immune protective efficacy of recombinant protein vaccines, but also to detect the vaccine-induced immune response and the effect on pathogen burden. Studies have shown that recombinant subunit vaccines based on major outer membrane proteins (MOMPs) bind CAF01 adjuvants to significantly reduce the loads of germ-borne pathogens by inducing Th1 immune responses, with a protective effect that depends on CD4^+^T cells (87). In terms of vaccine form innovation, transgenic rice expressing *MOMP* stimulates systemic IgG and mucosal sIgA antibodies through oral immunization, providing 50% protection against high-virulence strain challenge and significantly improving lung function (88) (**Figure 2**).

### 4.4 Limitations of translational research

The main limitations of natural host models lie in the high experimental costs and complex ethical considerations. Poultry models require high-level biosafety conditions, and the longer reproductive cycles of sheep and cattle models all limit the scale and reproducibility of the experiments. In addition, the complexity of the genetic background of the natural host also increases the difficulty of mechanistic studies.

Non-natural host models face restrictions on translational value brought about by species differences. The immune system of the mouse model differs significantly from that of humans, and the infection process in the guinea pig model cannot fully replicate the disease process in humans. Animal models such as pigs and horses, although physiologically closer to humans, lack genetic manipulation tools that constrain detailed molecular studies.

## 5 Discussion

Systematic comparison of animal models of *C. psittaci* shows that no single model can fully simulate the whole picture of human infection, especially in terms of reproductive tract structure, endocrine environment and the overall immunopathological process, given the wide host range and complex pathogenic mechanism of *C. psittaci*. Therefore, the selection of models must strictly serve the specific scientific problem, and the advancement of research depends on the integration of cross-model insights and technological innovation.

For example, the immunophenotypic differences between C57BL/6 and BALB/c mice are not merely a matter of experimental convenience but rather reflect profound and complex genetic determinants that dictate the interaction between the host and the pathogen. The C57BL/6 mice exhibit a strong Th1 bias and can promote rapid pathogen clearance mediated by IFN-γ, making them highly suitable for dissecting the cellular immune mechanism and resistance (37, 38). Based on this, the research on genetically modified mice has clearly demonstrated the crucial role of this cytokine in numerous aspects, and its effects are often achieved through multiple pathways (34, 89–91). In contrast, the BALB/c strain that is inclined towards the Th2 type exhibited more pronounced immunopathological changes and chronic infection symptoms, providing a unique perspective for studying vaccine-related enhanced diseases and their persistence mechanisms (32).

Although mouse models are highly effective in simplifying mechanism studies, their translational value is limited by significant species-specific differences in immune system structure and disease progression. In this case, non-human primate models become crucial. Their high similarity to humans in evolution and physiology enables them to accurately replicate key features of severe psittacosis, such as systemic inflammation and pneumonia, especially in cases of immune aging (92). Therefore, non-human primate models are suitable for validating findings from mouse studies and evaluating the efficacy and safety of major vaccine candidates or therapeutic drugs under conditions as close as possible to human infection.

To advance the research on *C. psittaci*, the following two major directions need to be focused on in the future. 1) Standardize the infection processes and detection methods of various models to facilitate effective comparisons and data integration among different studies, such as cytokine storm indices (93, 94). 2) Actively integrate cutting-edge technologies, such as using organoids to simulate the human mucosal barrier, or employing humanized mouse with *C. psittaci* specific HLA to analyze human-specific immune responses, thereby overcoming the barriers caused by species differences in transformation (95). In summary, only by systematically integrating natural and non-natural host models and collaborating with innovative technology platforms can we deeply reveal the immune pathogenic mechanism of *C. psittaci* and accelerate the development process of effective prevention and treatment strategies.
